# Risk Factors for Cognitive Impairment and Dementia Differ Across Classification Codes Evaluating Disadvantaged and Rural Regions in a Kentucky Appalachian Cohort

**DOI:** 10.1002/alz70860_106294

**Published:** 2025-12-23

**Authors:** Lauren Bojarski, Cassity High, Christopher J McLouth, Erin L. Abner, Rada Choate, Elif Pinar Coskun, Elizabeth K. Rhodus, Lauren Whitehurst, Frederick A Schmitt, Linda Jo Van Eldik, Gregory A Jicha

**Affiliations:** ^1^ University of Kentucky College of Medicine Department of Neurology, Lexington, KY, USA; ^2^ University of Kentucky College of Medicine Sanders‐Brown Center on Aging, Lexington, KY, USA; ^3^ University of Kentucky College of Medicine, Lexington, KY, USA; ^4^ University of Kentucky College of Medicine Department of Biostatistics, Lexington, KY, USA; ^5^ Department of Epidemiology and Environmental Health, University of Kentucky, Lexington, KY, USA; ^6^ University of Kentucky College of Public Health, Lexington, KY, USA; ^7^ University of Kentucky, SBCoA, Lexington, KY, USA; ^8^ University of kentucky, Department of Neurology, Lexington, KY, USA; ^9^ University of Kentucky College of Medicine Department of Behavioral Science, Lexington, KY, USA; ^10^ University of Kentucky College of Arts and Sciences Department of Psychology, Lexington, KY, USA; ^11^ University of Kentucky College of Medicine Department of Neurosciences, Lexington, KY, USA; ^12^ University of Kentucky Sanders‐Brown Center on Aging, Lexington, KY, USA

## Abstract

**Background:**

Older adults living in “rural” regions have an increased risk for developing impaired cognition (both cognitive impairment and dementia) based on risk factors such as reduced medical care access, transportation burden, lower socioeconomic position, higher rates of chronic conditions, etc. While “rural” classification is a recognized indicator of under‐represented group (URG) status by NIH definitions, there is no standard definition of rurality designated for use in research which complicates this knowledge. A comparison of known risk factors for cognitive decline/dementia and existing classification systems was done to analyze under‐represented groups and disadvantaged status in “rural” persons.

**Method:**

790 active University of Kentucky Alzheimer's Disease Research Center Cohort participants in 2024 were identified for this cross‐sectional, descriptive analysis. Health Resources and Services Administration (HRSA), Medically Underserved Areas/Health Professional Shortage Areas (MUA/HPSA), Area Deprivation Index (ADI) State Decile (cutoff set at ≥ 7th decile), ADI National Percentile scores (cutoff set at ≥70%), and Rural‐Urban Continuum Code (RUCC; cutoff set at ≥4) classifications were used to describe participants based on demographic information provided. These classifications were then analyzed with participant demographics including ApoE4 status and 15 medical conditions relative to cognitive impairment to evaluate relative risk against prevalence of cognitive impairment within the cohort.

**Result:**

Diagnostic classifications, mean age, and education did not differ significantly across classifications. Risks for tobacco use, alcohol use, traumatic brain injury, cardiovascular risks, stroke or transient ischemic attack, diabetes, hypertension, hyperlipidemia, vitamin B12 deficiency, hypothyroidism, sleep disturbances, depression, anxiety, cancer and chronic kidney disease were variably distributed across classifications. HRSA Rural and MUA/HPSA, as well as ADI National percentiles were associated with higher mean relative risk (∼1.2) and additive relative risk (2.3 to 3.6) profiles, whereas ADI State deciles and RUCC scores were not predictive of increased medical risks for cognitive impairment and dementia (RR<1.0).

**Conclusion:**

These data demonstrate that different classification systems designed to identify disadvantaged populations have different risk profiles for cognitive impairment and dementia. Understanding “rurality” and disadvantaged classification systems is complex but remains very important for research designed to identify high risk URG populations for cognitive decline & dementia.

